# Incident heart failure in patients with screening-detected atrial fibrillation: a *post hoc* analysis of the STROKESTOP and STROKESTOP II studies

**DOI:** 10.1093/europace/euag250

**Published:** 2026-09-03

**Authors:** Gina Sado, Carl Bonander, Katrin Kemp Gudmundsdottir, Catarina Djupsjö, Viveka Frykman Kull, Johan Engdahl, Emma Svennberg

**Affiliations:** Department of Medicine, Karolinska Institutet, Karolinska University Hospital Huddinge, SE-141 83 Huddinge, Sweden; Department of Cardiology, Danderyd University Hospital, SE-182 88 Stockholm, Sweden; School of Public Health and Community Medicine, Institute of Medicine, University of Gothenburg, Box 453, SE-405 30 Gothenburg, Sweden; Karlstad Business School, Karlstad University, SE-651 88 Karlstad, Sweden; Department of Clinical Sciences, Danderyd University Hospital, Karolinska Institutet, SE-182 88 Stockholm, Sweden; Division of Cardiac, Oncology and Ophthalmic Diseases, Department of Cardiology, Landspitali University Hospital, Hringbraut, 101 Reykjavik, Iceland; Department of Medicine, Karolinska Institutet, Karolinska University Hospital Huddinge, SE-141 83 Huddinge, Sweden; Department of Cardiology, Danderyd University Hospital, SE-182 88 Stockholm, Sweden; Department of Clinical Sciences, Danderyd University Hospital, Karolinska Institutet, SE-182 88 Stockholm, Sweden; Department of Cardiology, Danderyd University Hospital, SE-182 88 Stockholm, Sweden; Department of Clinical Sciences, Danderyd University Hospital, Karolinska Institutet, SE-182 88 Stockholm, Sweden; Department of Medicine, Karolinska Institutet, Karolinska University Hospital Huddinge, SE-141 83 Huddinge, Sweden

**Keywords:** Atrial fibrillation, Heart failure, Screening, STROKESTOP, STROKESTOP II, NT-proBNP

## Abstract

**Aims:**

Atrial fibrillation is associated with increased risks of stroke, heart failure, and death. While stroke prevention is a key focus, heart failure is more frequent and a major contributor to mortality in atrial fibrillation. This study aimed to assess the incidence and timing of heart failure in screening-detected atrial fibrillation.

**Methods and results:**

This *post hoc* analysis included participants without prior heart failure from the STROKESTOP and STROKESTOP II randomized screening studies, in which individuals aged 75–76 years were invited to ECG-based atrial fibrillation screening. Incident heart failure diagnoses were obtained from national registries. Incidence rates were calculated per 100 person-years, and Cox regression was used to estimate hazard ratios (HRs). In STROKESTOP, 23% of participants with screening-detected atrial fibrillation developed heart failure (3.76 per 100 person-years), compared with 4.43 in patients with known atrial fibrillation and 1.08 in patients without atrial fibrillation. Screening-detected atrial fibrillation was associated with a three-fold higher heart failure risk vs. no atrial fibrillation (adjusted HR 3.19) and a risk comparable to known atrial fibrillation (adjusted HR 2.86). In STROKESTOP II, 20% developed heart failure (4.19 per 100 person-years), compared with 3.52 in known atrial fibrillation and 0.93 without atrial fibrillation. Screening-detected atrial fibrillation was associated with a four-fold higher heart failure risk vs. no atrial fibrillation (adjusted HR 4.73) and a higher risk than known atrial fibrillation (adjusted HR 2.59).

**Conclusion:**

Screening-detected atrial fibrillation is associated with a high risk of heart failure, comparable to known atrial fibrillation, and is not a benign condition.

## Graphical Abstract

**Graphical Abstract:**
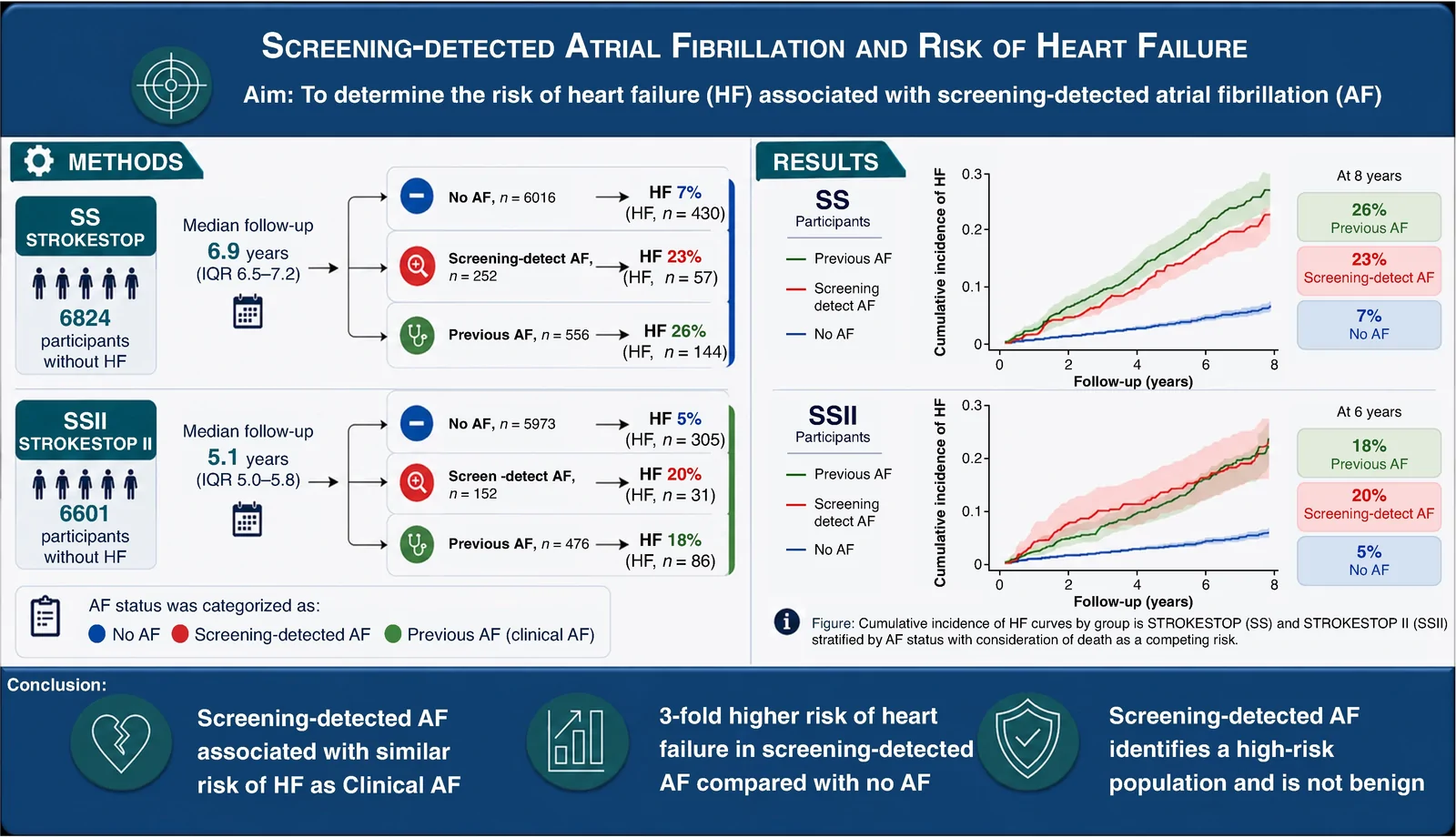

What’s new?

- The risk of heart failure in individuals with screening-detected atrial fibrillation is not well established.
- In participants without prior heart failure from two large screening studies (STROKESTOP and STROKESTOP II), screening-detected atrial fibrillation was associated with a risk of incident heart failure comparable to that of clinically diagnosed atrial fibrillation. Screening-detected atrial fibrillation was associated with a three- to four-fold higher risk of heart failure compared with individuals without atrial fibrillation.
- These findings indicate that screening-detected atrial fibrillation is not benign and support early cardiac evaluation and preventive strategies.

## Introduction

Atrial fibrillation (AF) is one of the most common arrhythmias, and the prevalence continues to increase globally.^1^ The condition represents a significant health and socioeconomic challenge, as it is associated with an increased risk of stroke, heart failure (HF), and death.^2, 3^ Due to its asymptomatic and intermittent character, AF may remain undetected and therefore untreated.^4^ Several screening studies, including the STROKESTOP studies^5, 6^ and the LOOP study,^7^ have evaluated the clinical benefit in randomized screening programmes for AF. These programmes have shown that screening could identify more patients with AF, and meta-analyses^8, 9^ suggest a reduction in stroke incidence.

Although stroke is a feared outcome in patients with AF, HF is more common^10^ and a major cause of death in these patients,^11^ yet it has received less attention than stroke. In patients with symptomatic AF, the risk of HF is well established.^12^ However, the risk of HF in screening-detected AF, which often is asymptomatic, is still not well understood.

In addition, patients with HF have a higher risk of developing AF.^13–15^ AF is present in ∼30–40% of patients with HF, and the two conditions frequently coexist and accelerate each other’s progression.^16, 17^ In patients with both AF and HF, AF has been associated with worsening HF and is an independent risk factor for all-cause mortality.^18^ As novel therapies emerge in patients with HF, early detection and treatment of HF might provide a prognostic benefit.^19^ To identify AF patients at increased risk of HF, clinical risk scores and biomarkers have been suggested.^20–22^

The aim of this study was to determine the incidence and timing of HF in participants with screening-detected AF compared with participants with previously known AF and those without AF.

## Methods

This study is a *post hoc* sub-analysis of the STROKESTOP and STROKESTOP II studies. Details and results of the studies have been published elsewhere.^2, 5, 6, 23^

In summary, the STROKESTOP study^2^ included all 75–76-year-old individuals living in Stockholm County (Sweden) or in the Halland Region of southwest Sweden. The study population in Stockholm was randomized 1:1 into two groups, either a control group or an intervention group. The intervention group consisted of *n* = 14 387 individuals.

STROKESTOP II^6, 23^ included all 75–76-year-old individuals living in the Stockholm Region (Sweden) (*n* = 28 712). The individuals were randomized 1:1 to either the control group or the intervention group (*n* = 14 356 in each group). In STROKESTOP II, all participants in the intervention group without a diagnosis of AF had N-terminal pro-B-type natriuretic peptide (NT-proBNP) analysed by venous blood samples using point-of-care analysis (Cobas 232, Roche Diagnostics, Rotkreuz, Switzerland). Depending on the result of NT-proBNP, the individuals were stratified into either a low-risk group (NT-proBNP <125 ng/L) or a high-risk group (NT-proBNP >125 ng/L).

In both studies, the participants without known AF in the intervention group performed an index ECG using a hand-held, single-lead device (Zenicor II, Zenicor, Stockholm, Sweden). If the index ECG showed sinus rhythm in the STROKESTOP study, participants performed ambulatory ECG recordings twice daily over a 2-week period (30 s measurements) and whenever they had symptoms. If the index ECG showed sinus rhythm in STROKESTOP II, the low-risk group with NT-proBNP <125 ng/L underwent no further investigation. Individuals in the high-risk group with NT-proBNP >125 ng/L performed intermittent, ambulatory ECG recordings four times daily. Control groups were not screened. Individuals with newly detected AF or with elevated NT-proBNP >900 ng/L were referred to a cardiologist for assessment.

Participants and controls from STROKESTOP and STROKESTOP II without a history of HF were evaluated according to AF status at baseline and incidence of HF. AF and HF diagnoses were recorded from registry data to preserve comparability with unscreened controls. In both studies, participants were categorized as having no AF (if no previous record of AF), previous AF (record of known diagnosis of AF), or screening-detected AF (AF detected during the screening programme). Controls were categorized as having no AF or previous AF. Individuals with registry-recorded HF at baseline were excluded from the analysis.

Outcome data, including HF defined by ICD code I50, baseline characteristics, baseline AF status defined by ICD code I48, and mortality data were obtained from national registries, including the National Patient Register, the National Cause of Death Register, and the Prescribed Drug Register. HF was defined as a diagnosis of I50 or the following ICD codes: I11.0, I13.0, I13.2, I25.5, I42, and I43, to capture patients with HF or related cardiac conditions not coded as I50.

The index date for screening participants was defined as the date of the screening visit. Controls were assigned index dates drawn at random from the empirical distribution of screening dates among participants. Follow-up for incident HF began at index and continued until HF diagnosis, death, emigration, or end of follow-up, whichever came first. Individuals with a recorded HF diagnosis prior to index were excluded.

AF status was classified as a fixed baseline exposure based on information available before and during the screening window (up to 2 weeks after index in STROKESTOP and up to 4 weeks after index in STROKESTOP II). Prior AF was defined as registry-recorded AF before index (present in both screened and control populations). Among individuals without prior AF, screening-detected AF was defined as AF identified during the screening window and assigned at index rather than at the exact date of detection (only present in screened populations). Individuals without prior or screening-detected AF constituted the reference group (no AF). These three mutually exclusive groups, no AF, screening-detected AF, and prior AF, formed the primary exposure classification in all analyses.

### Statistical analyses

Categorical variables were compared using the χ test. Continuous variables were analysed using analysis of variance. Cox proportional hazards models were used to estimate the risk of HF across patient groups, adjusting for predefined clinical variables to account for potential confounding. Fine–Gray subdistribution hazard models were used, with death from other causes as a competing risk.

The primary analysis used a fixed baseline AF classification reflecting the original STROKESTOP screening design, in which AF status was determined based on whether AF was detected at any point during the screening window. As a sensitivity analysis, we also fitted Cox models where AF was modelled as a time-varying exposure. In this analysis, individuals contributed unexposed person-time until their first AF diagnosis and exposed person-time thereafter, allowing registry-recorded AF diagnoses during follow-up to update exposure status over time. To preserve temporal ordering, an AF diagnosis was required to precede an HF event by at least 1 day to count as exposure. Screening-detected AF remained fixed at index, as screening was confined to the screening window.

As a separate sensitivity analysis, individuals with incident HF within 1 month of index were excluded to assess the potential impact of temporal misclassification arising from assignment of screening-detected AF to index rather than to the exact date of detection.

Results are presented as incidence rates and hazard ratios (HRs) with 95% confidence intervals (CIs). All statistical analyses were performed using R (R Foundation for Statistical Computing, Vienna, Austria).

### Ethics

STROKESTOP and STROKESTOP II comply with the Declaration of Helsinki. The protocol was approved by the regional ethics committee (STROKESTOP DNR 2011-1363-31/3, STROKESTOP II DNR 2015/2079-31/1). Written informed consent was obtained from all participants in the intervention group.

## Results

### Study population

**Figure 1:**
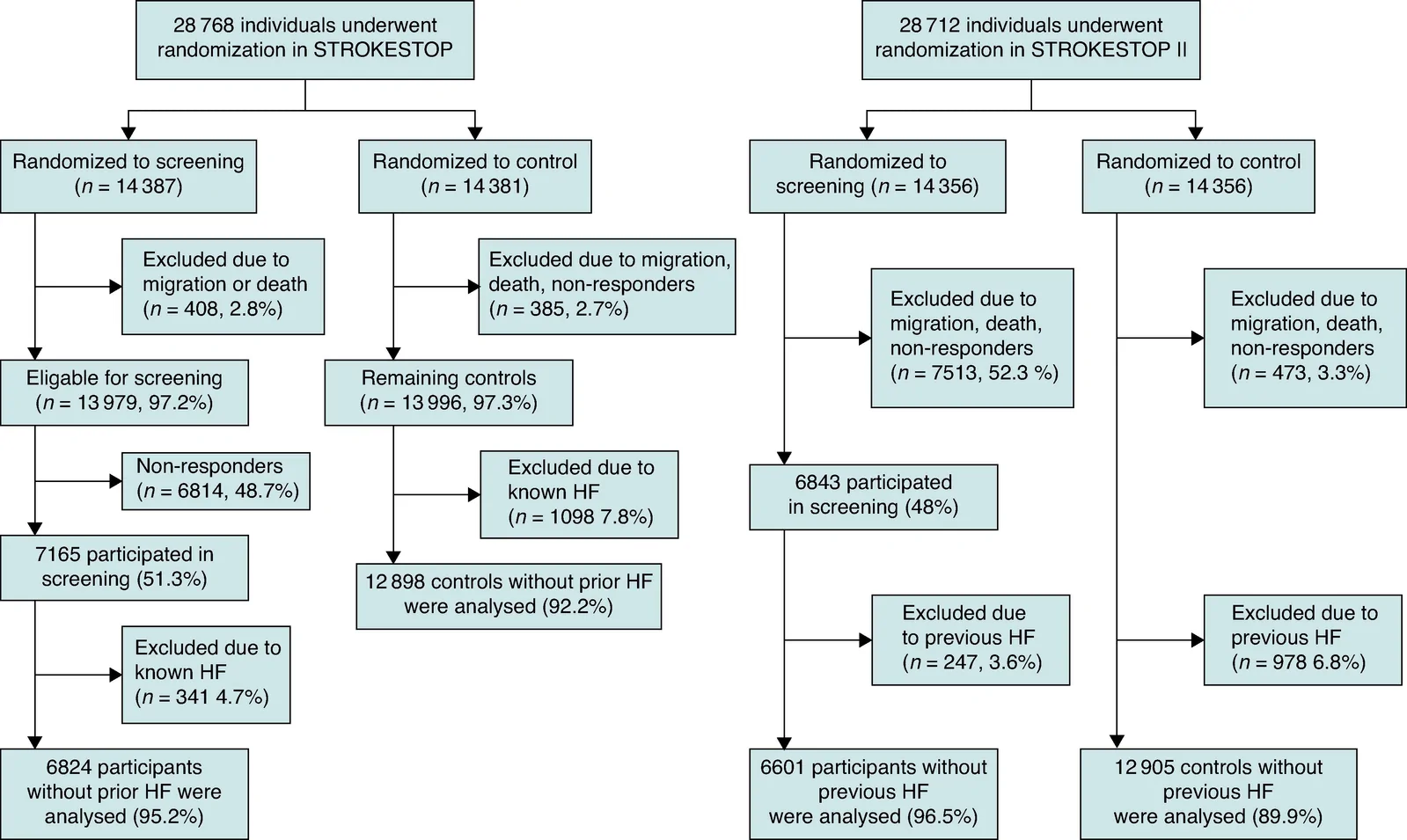
Flow chart of the study population in STROKESTOP and STROKESTOP II without previous HF. HF, heart failure; AF, atrial fibrillation.

In the STROKESTOP study, 7165 participants were screened for AF from 2012 to 2014. After exclusion of participants with previous HF (*n* = 341), a total of 6824 participants were included in this sub-study and analysed according to AF status (no AF, previous AF, or screening-detected AF) and incident HF. A total of 12 898 controls were included after excluding 1098 participants with previous HF.^5^ A detailed flow chart of the study population is shown in *Figure 1*.

In the STROKESTOP II study, 6843 participants were screened for AF from 2016 to 2018. A total of 6601 participants in the screening group were analysed concerning AF status and incidence HF, after excluding 247 individuals due to known HF.^6^ In the control group, a total of 12 905 individuals were included for analysis after excluding those with previous HF (*n* = 978). A detailed flow chart of the study population is shown in *Figure 1*.

### Baseline characteristics

In the STROKESTOP study, 3.7% (252/6824) of the participants had screening-detected AF, 8.1% (556/6824) had previously known AF, and 88.2% (6016/6824) had no AF diagnosis in registries. Among controls in STROKESTOP, 9.6% (1232/12 898) had known AF and 90.4% (11 666/12 898) had no AF.

Baseline characteristics stratified by AF status in STROKESTOP are presented in *Table 1*. Participants with previously known AF were older, more often male, and had a higher burden of cardiovascular comorbidities and more medication use compared with those without AF and those with screening-detected AF. Hypertension, diabetes, coronary heart disease, prior stroke or transient ischaemic attack, and other vascular disease were more prevalent in this group, resulting in higher CHA_2_DS_2_-VASc scores.

**Table 1:** Baseline characteristics of participants in STROKESTOP without prior HF stratified by AF status

	No AF	Previous AF	Screening-detected AF	*P*-value
	(*n* = 6016)	(*n* = 556)	(*n* = 252)	
Age, mean years (SD)	75.8 (0.74)	75.94 (0.74)	75.87 (0.75)	<0.001
Female, *n* (%)	3392 (56.4)	222 (39.9)	117 (46.4)	<0.001
Living alone, *n* (%)	2728 (45.3)	231 (41.5)	112 (44.4)	0.223
Household in highest income quartile, *n* (%)	1889 (31.4)	192 (34.5)	77 (30.6)	0.294
University education, *n* (%)	2100 (34.9)	185 (33.3)	75 (29.8)	0.193
Alcohol-related disorder, *n* (%)	66 (1.1)	8 (1.4)	2 (0.8)	0.676
CHA2DS2-VASc score (categorical), *n* (%)				<0.001
Only age and sex	3839 (63.8)	148 (26.6)	138 (54.8)	
1–2 additional risk factors	1863 (31.0)	301 (54.1)	93 (36.9)	
3 or more additional risk factors	314 (5.2)	107 (19.2)	21 (8.3)	
Diabetes, *n* (%)	587 (9.8)	102 (18.3)	42 (16.7)	<0.001
Hypertension, *n* (%)	1592 (26.5)	348 (62.6)	85 (33.7)	<0.001
Coronary heart disease, *n* (%)	559 (9.3)	131 (23.6)	33 (13.1)	<0.001
Prior stroke/TIA, *n* (%)	325 (5.4)	101 (18.2)	21 (8.3)	<0.001
Other vascular disease, *n* (%)	114 (1.9)	27 (4.9)	8 (3.2)	<0.001
Renal disease, *n* (%)	37 (0.6)	10 (1.8)	1 (0.4)	0.005
Dementia, *n* (%)	49 (0.8)	11 (2.0)	1 (0.4)	0.014
Cancer in last 3 years, *n* (%)	717 (11.9)	98 (17.6)	29 (11.5)	<0.001
Intracardiac thrombus/embolism, *n* (%)	3 (0.0)	1 (0.2)	0 (0.0)	0.445
Arterial embolism, *n* (%)	22 (0.4)	4 (0.7)	1 (0.4)	0.446
Venous thromboembolism, *n* (%)	263 (4.4)	41 (7.4)	12 (4.8)	0.006
ACE inhibitor/ARB, *n* (%)	2013 (33.5)	254 (45.7)	99 (39.3)	<0.001
Aspirin, *n* (%)	1273 (21.2)	134 (24.1)	69 (27.4)	0.021
Beta-blocker, *n* (%)	1419 (23.6)	389 (70.0)	79 (31.3)	<0.001
Statins, *n* (%)	1549 (25.7)	222 (39.9)	78 (31.0)	<0.001
Antiplatelet agents, *n* (%)	1366 (22.7)	145 (26.1)	74 (29.4)	0.012
MRA, *n* (%)	61 (1.0)	14 (2.5)	6 (2.4)	0.002
Digitalis glycosides, *n* (%)	0 (0.0)	62 (11.2)	2 (0.8)	<0.001
Diuretics, *n* (%)	890 (14.8)	135 (24.3)	53 (21.0)	<0.001
Long-acting nitrates, *n* (%)	110 (1.8)	23 (4.1)	8 (3.2)	0.001
Calcium-channel blocker, *n* (%)	1168 (19.4)	151 (27.2)	55 (21.8)	<0.001

Heparin and aspirin were excluded from the antiplatelet agents group. Other vascular disease: peripheral arterial disease.

HF, heart failure; AF, atrial fibrillation; TIA, transient ischaemic attack; ACE inhibitor, angiotensin-converting enzyme inhibitor; ARB, angiotensin II receptor blocker; MRA, mineralocorticoid receptor antagonist; SD, standard deviation; IQR, interquartile range.

In STROKESTOP II, 2.3% (152/6601) participants had screening-detected AF, 7.2% (476/6601) had previously known AF, and 90.5% (5973/6601) had no AF. Among controls in STROKESTOP II, 10.2% (1313/12 905) had previously known AF and 89.8% (11 592/12 905) had no AF. Baseline characteristics stratified by AF status are shown in *Table 2*. Participants with previously known AF were older, more often male, and had a higher prevalence of cardiovascular comorbidities and medication use compared with the other groups. NT-proBNP levels in participants with known AF and screening-detected AF were higher than in participants with no AF.

**Table 2:** Baseline characteristics of participants in STROKESTOP II without prior HF stratified by AF status

	No AF	Previous AF	Screening-detected AF	*P*-value
	(*n* = 5973)	(*n* = 476)	(*n* = 152)	
Age, mean years (SD)	76.12 (0.55)	76.18 (0.55)	76.07 (0.50)	0.044
Female, *n* (%)	3349 (56.1)	184 (38.7)	72 (47.4)	<0.001
Living alone, *n* (%)	2540 (42.5)	162 (34.0)	71 (46.7)	0.001
Disposable income >300 000 SEK/yr, *n* (%)	1315 (22.0)	131 (27.5)	39 (25.7)	0.014
University education, *n* (%)	2438 (40.8)	190 (39.9)	66 (43.4)	0.746
Alcohol-related disorder, *n* (%)	118 (2.0)	9 (1.9)	6 (3.9)	0.228
CHA2DS2-VASc score (categorical), *n* (%)				<0.001
Only age and sex	3563 (59.7)	129 (27.1)	91 (59.9)	
1–2 additional risk factors	2049 (34.3)	278 (58.4)	56 (36.8)	
3 or more additional risk factors	361 (6.0)	69 (14.5)	5 (3.3)	
Diabetes, *n* (%)	673 (11.3)	78 (16.4)	14 (9.2)	0.002
Hypertension, *n* (%)	1872 (31.3)	287 (60.3)	54 (35.5)	<0.001
Coronary heart disease, *n* (%)	555 (9.3)	99 (20.8)	11 (7.2)	<0.001
Prior stroke/TIA, *n* (%)	214 (3.6)	57 (12.0)	3 (2.0)	<0.001
Other vascular disease, *n* (%)	101 (1.7)	13 (2.7)	1 (0.7)	0.146
Renal disease, *n* (%)	78 (1.3)	17 (3.6)	1 (0.7)	<0.001
Dementia, *n* (%)	63 (1.1)	9 (1.9)	2 (1.3)	0.243
Cancer in last 3 years, *n* (%)	903 (15.1)	78 (16.4)	25 (16.4)	0.696
Intracardiac thrombus/embolism, *n* (%)	2 (0.0)	0 (0.0)	0 (0.0)	0.900
Arterial embolism, *n* (%)	17 (0.3)	3 (0.6)	1 (0.7)	0.328
Venous thromboembolism, *n* (%)	350 (5.9)	42 (8.8)	18 (11.8)	0.001
ACE inhibitor/ARB, *n* (%)	2338 (39.1)	231 (48.5)	57 (37.5)	<0.001
Aspirin, *n* (%)	1032 (17.3)	63 (13.2)	18 (11.8)	0.019
Beta-blocker, *n* (%)	1253 (21.0)	331 (69.5)	36 (23.7)	<0.001
Statins, *n* (%)	1651 (27.6)	192 (40.3)	32 (21.1)	<0.001
Antiplatelet agents, *n* (%)	1154 (19.3)	71 (14.9)	19 (12.5)	0.008
MRA, *n* (%)	76 (1.3)	15 (3.2)	4 (2.6)	0.002
Digitalis glycosides, *n* (%)	2 (0.0)	29 (6.1)	0 (0.0)	<0.001
Diuretics, *n* (%)	701 (11.7)	70 (14.7)	19 (12.5)	0.155
Long-acting nitrates, *n* (%)	89 (1.5)	12 (2.5)	3 (2.0)	0.204
Calcium-channel blocker, *n* (%)	1170 (19.6)	115 (24.2)	28 (18.4)	0.050
NT-proBNP, median (IQR)	146 (82–251)	305(152.5–475.5)	322 (203.5, 683)	<0.001
BMI, mean (SD)	25.58 (3.88)	25.68 (4.43)	25.92 (4.18)	0.551

Heparin and aspirin were excluded from the antiplatelet agents group. Other vascular disease: peripheral arterial disease.

HF, heart failure; AF, atrial fibrillation; TIA, transient ischaemic attack; ACE inhibitor, angiotensin-converting enzyme inhibitor; ARB, angiotensin II receptor blocker; MRA, mineralocorticoid receptor antagonist; NT-proBNP, N-terminal pro-B-type natriuretic peptide; BMI, body mass index; SD, standard deviation; IQR, interquartile range.

### Incidence of HF in screening-detected AF

In the STROKESTOP study, HF developed in 23% of participants with screening-detected AF (57/252), 26% of participants with previously known AF (144/556), and 7% of participants without AF (430/6016) after a median follow-up of 6.9 years (IQR 6.5–7.2). Among controls, 26% of those with previously known AF (322/1232) and 9.4% of those without AF (1107/11 666) developed HF.

In STROKESTOP, participants with screening-detected AF had an incidence rate that was similar to that observed among individuals with previously known AF (3.76 vs. 4.43 per 100 person-years). The lowest HF incidence rate among participants was observed in individuals without AF. Among controls, the highest HF incidence was observed in individuals with previously known AF (4.91 per 100 person-years), while the lowest rates were seen in individuals without AF. Details concerning incidence rates are shown in *Table 3*.

**Table 3:** HF incidence rates and hazard ratios according to AF status in STROKESTOP and STROKESTOP II participants

SS						
Outcome	Group	Events (HF)	Person-years	IR per 100 PY (95% CI)	HR (95% CI)	Adjusted HR (95% CI)
HF	No AF (ref) (*n* = 6016)	430	39 945	1.08 (0.98–1.18)	1.00	1.00
HF	Screening-detected AF (*n* = 252)	57	1516	3.76 (2.85–4.87)	3.58 (2.71–4.72)	3.19 (2.42–4.21)
HF	Previous AF (*n* = 556)	144	3248	4.43 (3.74–5.22)	4.27 (3.53–5.15)	2.86 (2.34–3.50)
**SS II**						
**Outcome**	**Group**	**Events (HF)**	**Person-years**	**IR per 100 PY (95% CI)**	**HR (95% CI)**	**Adjusted HR (95% CI)**
HF	No AF (ref) (*n* = 5973)	305	32 900	0.93 (0.83–1.04)	1.00	1.00
HF	Screening-detected AF (*n* = 152)	31	741	4.19 (2.84–5.94)	4.58 (3.16–6.63)	4.73 (3.26–6.85)
HF	Previous AF (*n* = 476)	86	2441	3.52 (2.82–4.35)	3.86 (3.04–4.90)	2.59 (2.01–3.33)

SS/SSII adjusted models include age, female gender, coronary heart disease, hypertension, diabetes, previous stroke/TIA, chronic kidney disease, cancer, vascular disease, and valvular heart disease.

SS, STROKESTOP; SSII, STROKESTOP II; HF, heart failure; AF, atrial fibrillation; PY, person-years; CI, confidence interval; IR, incident rate; HR, hazard ratio; ref, reference group.

In STROKESTOP II, after a median follow-up of 5.1 years (IQR 5.0–5.8), HF developed in 20% of participants with screening-detected AF (31/152), 18% of participants with previously known AF (86/476), and 5% of participants without AF (305/5973). Among controls, HF occurred in 22% of those with previously known AF (286/1313) and 6.4% of those without AF (764/11 592).

**Figure 2:**
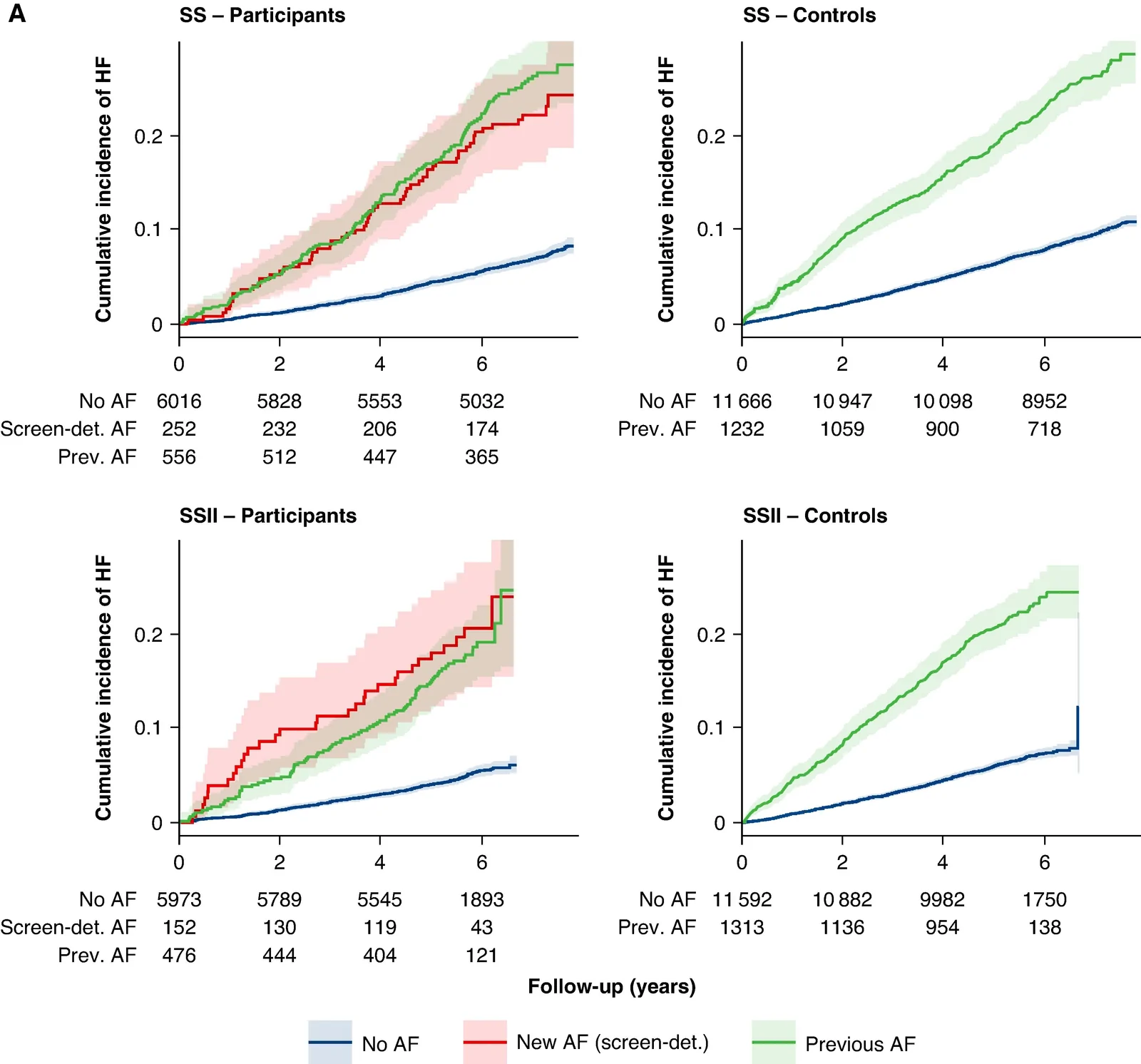

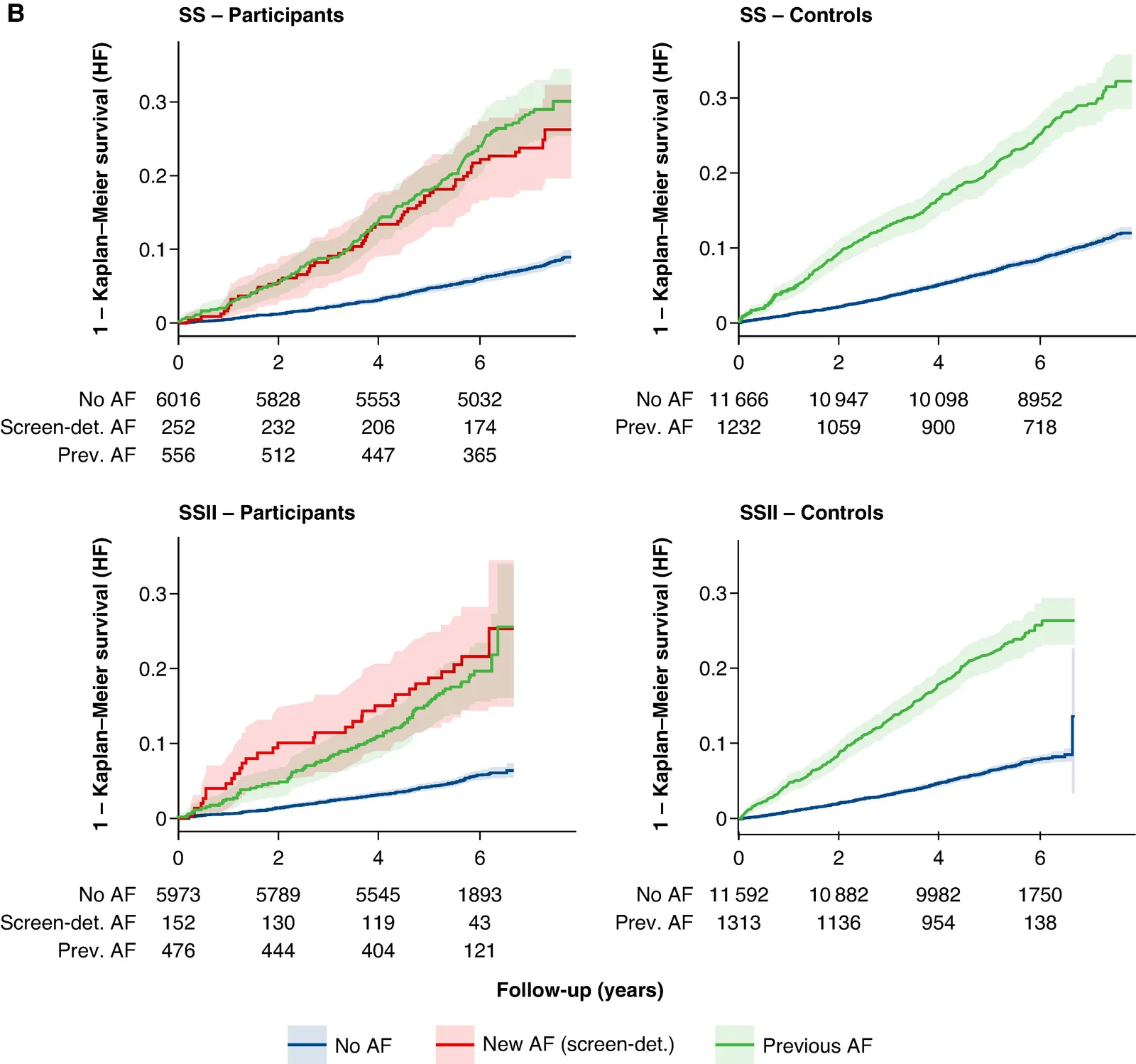
(*A*) Cumulative incidence of HF curves by group in STROKESTOP (SS) and STROKESTOP II (SSII) stratified by AF status with consideration of death as a competing risk. Numbers below each subfigure refer to the number of individuals at risk. HF, heart failure; AF, atrial fibrillation; screen-det., screening-detected. (*B*) Kaplan–Meier estimates of incident HF by group in STROKESTOP (SS) and STROKESTOP II (SSII). Numbers below each subfigure refer to the number of individuals at risk. Individuals who died during follow-up were censored. HF; heart failure, AF; atrial fibrillation; screen-det., screening-detected.

In STROKESTOP II, incidence rates were similar to those observed in STROKESTOP (*Table 3*). Participants with screening-detected AF had an HF incidence comparable to those with previously known AF (4.19 vs. 3.52 per 100 person-years), although numerically higher, while the lowest rates were observed among participants without AF. Among controls, the highest incidence rate was observed in those with previously known AF compared with controls without AF (4.90 vs. 1.32 per 100 person-years). Incidence rates were further illustrated using cumulative incidence and Kaplan–Meier curves (*Figure 2A* and *B*), showing that most HF diagnoses occurred approximately within 6 months across both studies and groups.

In STROKESTOP II, NT-proBNP levels were analysed in participants who underwent screening (see Supplementary material online, *Table S1*). In participants with screening-detected AF who developed HF, NT-proBNP levels were higher (median NT-proBNP 614.5 ng/L, IQR 335.5–1276.5) compared with participants without AF who developed HF (median NT-proBNP 261 ng/L, IQR 154.3–478.5). NT-proBNP levels among participants with screening-detected AF and HF were comparable to those observed among participants with previously known AF and HF (median 603.5 ng/L, IQR 352.8–783.0).

In the Cox regression analysis (*Tables 3* and *4*), screening-detected AF was consistently associated with a higher risk of HF, with risk estimates comparable to and, in some models, exceeding those for previously known AF. Both participants with previous AF and screening-detected AF were associated with a higher risk of incident HF compared with participants without AF in both STROKESTOP and STROKESTOP II. The adjusted HRs in STROKESTOP were 3.19 (95% CI, 2.42–4.21) for screening-detected AF and 2.86 (95% CI, 2.34–3.50) for previously known AF. The corresponding adjusted HRs in STROKESTOP II were 4.73 (95% CI, 3.26–6.85) and 2.59 (95% CI, 2.01–3.33), respectively. Further adjustment for NT-proBNP, height, and weight attenuated the association for previous AF, but it remained significant in screening-detected AF (see supplementary material online, *Tables S2A* and *B*).

**Table 4:** HF incidence rates and hazard ratios according to AF status in STROKESTOP and STROKESTOP II controls

SS						
Outcome	Group	Events (HF)	Person-years	IR per 100 PY (95% CI)	HR (95% CI)	Adjusted HR (95% CI)
HF	Controls, no AF (ref) (*n* = 11 666)	1107	71 730	1.54 (1.45–1.64)	1.00	1.00
HF	Controls, previous AF (*n* = 1232)	322	6563	4.91 (4.39–5.47)	3.25 (2.87–3.67)	2.36 (2.07–2.69)
**SSII**						
**Outcome**	**Group**	**Events (HF)**	**Person-years**	**IR per 100 PY (95% CI)**	**HR (95% CI)**	**Adjusted HR (95% CI)**
HF	Controls, no AF (ref) (*n* = 11 592)	764	57 716	1.32 (1.23–1.42)	1.00	1.00
HF	Controls, previous AF (*n* = 1313)	286	5832.9	4.9 (4.35–5.51)	3.75 (3.27–4.29)	2.65 (2.29–3.06)

SS/SSII adjusted models include age, female gender, coronary heart disease, hypertension, diabetes, previous stroke/TIA, chronic kidney disease, cancer, vascular disease, and valvular heart disease.

SS, STROKESTOP; SSII, STROKESTOP II; HF, heart failure; AF, atrial fibrillation; PY, person-years; CI, confidence interval; HR, hazard ratio; ref, reference group.

Controls with previously known AF had a significantly higher risk of developing incident HF in both STROKESTOP and STROKESTOP II compared with controls without AF (*Table 4*). The risk was approximately three- to four-fold higher in both studies in unadjusted models. After adjusting for sex, age, and cardiovascular comorbidities, the association remained but was attenuated (adjusted HRs of 2.36 in STROKESTOP and 2.65 in STROKESTOP II).

In Fine–Gray models accounting for competing risks, associations between AF status and incident HF were consistent with Cox models (see Supplementary material online, *Table S3A* and *B*). After adjustment, HRs remained elevated for screening-detected AF in both studies but were attenuated for previously known AF after additional adjustment for NT-proBNP, weight, and height in STROKESTOP II. Among controls (see Supplementary material online, *Table S3B*), previously known AF was associated with higher HF risk, which was attenuated after adjustment. Overall, Fine–Gray analyses supported a strong association between AF, particularly screening-detected AF, and subsequent HF.

In the time-varying AF sensitivity analysis, screening-detected AF and previous AF remained associated with higher rates of incident HF, and incident AF recorded during follow-up was also associated with higher rates of incident HF (see Supplementary material online, *Table S4*). In the sensitivity analysis excluding individuals with incident HF within 1 month of index, the estimates were similar to those in the primary analysis (see Supplementary material online, *Tables S5A* and *B*). Heart failure events within the 1-month post-index window, during which the exact date of screening-detected AF could not be determined, were uncommon among participants and were excluded in a sensitivity analysis: 4/6824 (0.06%) in STROKESTOP and 4/6601 (0.06%) in STROKESTOP II. The excluded events are summarized in Supplementary material online, *Table S5B*.

## Discussion

In this sub-study, we show that patients with screening-detected AF developed HF with a similar incidence rate as patients with known AF.

Our findings support the notion that screening-detected AF is not a benign condition. In this study, individuals with AF detected through intermittent monitoring had a significantly increased risk of developing HF, a risk comparable to that observed in patients with clinically diagnosed AF. Notably, in this analysis, screening-detected AF was associated with a three- to four-fold higher risk of incident HF, highlighting its clinical relevance. These results support the value of AF screening not only for early detection of arrhythmia but also as a potential gateway to earlier identification and management of HF. These findings further suggest that individuals with screening-detected AF may benefit from clinical and echocardiographic evaluation, similar to patients with clinically diagnosed AF, as recommended in the ESC guidelines, to facilitate timely diagnosis and treatment of underlying cardiac dysfunction. In addition, more long-term and repeated follow-up may be warranted in this group to improve early detection and management of HF.^16, 17^

The relationship between known AF and HF is well established.^24–26^ Approximately 30% of patients with newly detected AF have a history of HF, and patients with clinical AF are more likely to develop HF. The PREVEND trial (Prevention of Renal and Vascular End-stage Disease, *n* = 8265) included individuals without known AF who underwent repeated ECG screening over long-term follow-up, allowing identification of both screening and clinically detected AF.^27^ In PREVEND, no difference in incident HF was observed between screening and clinically detected AF, suggesting a similar prognosis regardless of detection method, which is consistent with our findings. It is probable that these participants with AF detected at a single time point were more likely to have persistent or permanent AF or a high AF burden.^27^

AF pattern matters for the development of HF, and prior studies have shown that patients with permanent AF have the highest risk of developing HF.^28^ The higher incidence of HF in long-lasting AF (persistent and permanent) could be explained by persistent tachycardia, leading to tachycardia-induced cardiomyopathy, but also by progressive structural remodelling, leading to atrial dilation, fibrosis, neurohormonal activity, ion-channel and cell remodelling, gap-junction disruption, and electrical heterogeneity in myocardial tissue. These atrial structural changes can impair diastolic filling and cause elevated left ventricular pressures and hence HF.^29, 30^ The lesser extent of these changes in paroxysmal AF may partly explain the lower incidence of HF in this subgroup.^29^ The concept of atrial cardiomyopathy, which includes structural and electrophysiological atrial alterations beyond AF itself, may represent an important pathophysiological link between screening-detected AF and incident HF.^31^ Screening-detected AF, like clinically known AF, may partly reflect underlying structural heart disease and/or atrial cardiomyopathy. Elevated NT-proBNP levels in this group further suggest the presence of subclinical cardiac dysfunction not fully captured in administrative registers. Rather than undermining the findings, these considerations suggest that screening-detected AF identifies a high-risk phenotype warranting close cardiovascular follow-up.

Additionally, beyond AF patterns, emerging evidence suggests that AF burden, defined as the proportion of time spent in AF, may influence HF risk independently of traditional AF pattern classification (permanent, persistent, paroxysmal), representing an important and underexplored determinant of cardiovascular outcomes, including HF.^32^ Even in patients with very low AF burden, such as those with device-detected AF, an association with adverse outcomes has been observed. Recent data from the ARTESiA trial suggest that progression of device-detected AF is associated with an increased risk of adverse outcomes, including HF-related death.^27, 33^ Similarly, in the LOOP study, implantable loop recorder (ILR)-detected AF (often characterized by low AF burden) was associated with an increased risk of HF, particularly in patients with episodes ≥24 h (HR 4.8, 95% CI 1.62–14.23).^30^ In our sub-study, we observed comparable hazard ratios in screening-detected AF in STROKESTOP (HR 3.58, 95% CI 2.71–4.72) and STROKESTOP II (HR 4.58, 95% CI 3.16–6.63). Notably, intermittent ECG screening over 14 days has limited sensitivity^2^ and is therefore more likely to identify individuals with a higher AF burden, hence providing a possible explanation for why screening-detected AF carries a risk comparable to clinically diagnosed AF. Future studies incorporating continuous AF burden quantification may provide further insight into the dose-response relationship between AF and HF risk.^32^

The findings of this study may have important implications from a health economic perspective. AF screening programmes have previously been evaluated in terms of cost-effectiveness, with variable estimates across different European countries.^34^ The recognition that screening-detected AF is associated with a substantially increased risk of subsequent HF adds a further dimension to these assessments. Whether early identification of high-risk individuals through screening, followed by appropriate cardiovascular follow-up and preventive strategies, could reduce HF-related healthcare costs warrants dedicated health economic evaluation. These considerations align with the emerging concept of value-based healthcare in cardiology and electrophysiology, which emphasizes the importance of evaluating screening interventions not only by their primary endpoint but by the broader value they offer to patients and health systems.^35^

NT-proBNP levels may further help identify which individuals with screening-detected AF are at highest risk of developing HF. Natriuretic peptides are elevated in both individuals with HF^36^ and in AF^37^ and have proven to be a predictor of incident AF.^38^ In STROKESTOP II, NT-proBNP was used to identify individuals who may benefit from AF screening.^23^ Consistent with findings from the LOOP study, higher NT-proBNP levels were associated with higher baseline stroke risk and may help identify individuals more likely to benefit from screening in both studies.^39^ In our sub-study, NT-proBNP levels were higher among participants with screening-detected AF who developed HF compared with participants without AF who developed HF. Our findings suggest that NT-proBNP may be useful not only as a selection tool for AF screening but also after inclusion to facilitate early detection of HF.

At present, we lack good strategies to predict and prevent incident HF in AF. Although the Framingham study conducted a risk score showing good risk discrimination, it has not been shown to be generalizable and performed inadequately in elderly patients.^21^ Risk scores incorporating clinical variables and biomarkers such as NT-proBNP, Troponin T, and GDF-15 have shown promise in identifying AF patients at high risk of HF hospitalization,^22, 40^ highlighting the potential for a combined biomarker and clinical risk stratification approach in this population. Similarly, in our study, higher NT-proBNP levels were observed in participants with screening-detected and previously known AF compared with those without AF (see Supplementary material online, *Table S1*), underscoring the potential of biomarkers in screening for both AF and HF.

### Study limitations

STROKESTOP and STROKESTOP II are two large, randomized screening trials that share a common limitation related to participation bias in the screened population. Previous studies have shown that individuals who participate in screening studies tend to be healthier and have a lower burden of disease compared with non-participants.^41–43^ Sub-studies from STROKESTOP and STROKESTOP II confirmed that non-participants had lower socioeconomic status, poorer health, and higher CHA_2_DS_2_-VASc scores than participants.^2, 23, 44, 45^ This selective participation may limit the generalizability of our findings from the screened participants to the broader population. Characteristics and outcomes of non-participants in relation to newly detected AF and HF were not assessed in this study.

The study consisted of 75–76-year-old individuals living in Sweden, which reduces the generalizability of the results in younger populations. As this was an observational analysis and the original study was not designed to assess HF, causal inferences cannot be made. As with all observational studies, residual confounding cannot be ruled out, and there are several factors we would have liked to measure that are not available in our data, including lifestyle factors, frailty, and subclinical cardiac dysfunction. Screening-detected AF, like clinically known AF, may partly reflect underlying structural heart disease or atrial cardiomyopathy, not fully captured by registry-based covariate adjustment.^31^

The number of participants in the screening-detected AF group was small, especially in STROKESTOP II, which could result in limited power in this group. Paroxysmal or low-burden AF may have remained undetected in all groups, particularly among controls, potentially contributing to selection and misclassification bias.

HF diagnosis was registry-based, with no differentiation between HF subtypes, limiting mechanistic interpretation, as ∼80% of HF diagnoses were coded as I50.9 (unspecified). ICD-10 subcodes for HF subtypes were not introduced in Sweden until 2021, covering only a minority of the follow-up period.^46^ Whether the observed association differs by ejection fraction phenotype, such as the predominantly heart failure with preserved ejection fraction (HFpEF) pattern (left ventrical ejection fraction [LVEF] >50%) and adverse outcomes reported in asymptomatic AF populations,^47^ therefore remains to be determined. Registry-based HF diagnosis may have led to missing HF cases due to imperfect diagnosis capture and incomplete representation of primary care in the National Patient Register.

Another limitation is that screening-detected AF was assigned to the index date rather than to the exact date of detection within the screening window. However, excluding individuals with HF within 1 month of index did not materially alter the results (see Supplementary material online, *Tables S5A* and *B*). NT-proBNP was only analysed in participants who underwent screening in STROKESTOP II, which prevented comparison with the control group.

Additionally, individuals with screening-detected AF may have more frequent subsequent healthcare contacts, including echocardiographic evaluation, which could potentially increase the likelihood of HF being diagnosed. This surveillance bias may have contributed to an overestimation of the observed associations.

Whether a systematic screening strategy for AF reduces the long-term risk of HF through earlier detection and treatment remains an important question for future research. Addressing this would ideally require a prospective study design with an intention-to-treat framework, where participants are analysed according to their assigned screening group regardless of whether AF was detected.

### Strengths

This study was based on two large population-based cohorts, which enhances external validity and generalizability to older individuals in the general population. Consistent findings of HF incidence observed in both STROKESTOP and STROKESTOP II strengthen the reliability of the results. Including both screened participants and controls allows comparison between screened and unscreened groups, which provides context for the observed incidence rates. Long-term follow-up and the use of nationwide health registries allowed us to capture most HF cases.

## Conclusion

Individuals with screening-detected AF have a similar risk of developing HF as those with clinically diagnosed AF and a three- to four-fold higher risk compared with participants without AF. These findings indicate that screening-detected AF is not a benign condition. The present findings reinforce that AF identified through screening carries clinically relevant cardiovascular risk comparable to previously known AF and should not be regarded as a less important entity.

## Supplementary Material

euag250_Supplementary_Data

## Data Availability

The data underlying this article cannot be shared publicly due to ethical and legal restrictions protecting the privacy of study participants. The data were collected under ethical approval and are governed by Swedish data protection regulations.
