# Supplementary material for: Incident heart failure in patients with screening-detected atrial fibrillation: a post hoc analysis of the STROKESTOP and STROKESTOP II studies
Source: Europace. 2026 Sep 3;28(9):euag250. doi: 10.1093/europace/euag250 (PMC13595287; doi:10.1093/europace/euag250)
Supplement: Supplementary file 1 [file euag250_supplementary_data.docx]

| **Supplementary Table S1. Participants with new HF diagnosis stratified by AF status in STROKESTOP II** | | | | | |
| --- | --- | --- | --- | --- | --- |
|  | No AF | Previous AF | New AF (screen det.) | p-value |  |
|  | (n=305) | (n=86) | (n=31) |  |  |
| Age, mean years (SD) | 76.12 (0.60) | 76.22 (0.56) | 76.13 (0.56) | 0.360 |  |
| Female, n (%) | 137 (44.9) | 38 (44.2) | 16 (51.6) | 0.756 |  |
| Living alone, n (%) | 145 (47.5) | 31 (36.0) | 14 (45.2) | 0.167 |  |
| Disposible income >300 000 SEK/yr, n (%) | 55 (18.0) | 18 (20.9) | 11 (35.5) | 0.066 |  |
| University education, n (%) | 96 (31.5) | 30 (34.9) | 18 (58.1) | 0.012 |  |
| Alcohol-related disorder,  n (%) | 9 (3.0) | 2 (2.3) | 3 (9.7) | 0.116 |  |
| CHA2DS2-VASc score (categorical), n (%) |  |  |  | <0.001 |  |
| Only age and sex | 116 (38.0) | 15 (17.4) | 17 (54.8) |  |  |
| 1-2 additional risk factors | 141 (46.2) | 50 (58.1) | 13 (41.9) |  |  |
| 3 or more additional risk factors | 48 (15.7) | 21 (24.4) | 1 (3.2) |  |  |
| Diabetes, n (%) | 65 (21.3) | 20 (23.3) | 2 (6.5) | 0.119 |  |
| Hypertension, n (%) | 161 (52.8) | 60 (69.8) | 11 (35.5) | 0.002 |  |
| Coronary heart disease,  n (%) | 67 (22.0) | 24 (27.9) | 5 (16.1) | 0.336 |  |
| Prior stroke/TIA, n (%) | 22 (7.2) | 16 (18.6) | 1 (3.2) | 0.003 |  |
| Other vascular disease, n (%) | 7 (2.3) | 2 (2.3) | 0 (0.0) | 0.694 |  |
| Renal disease, n (%) | 11 (3.6) | 4 (4.7) | 0 (0.0) | 0.485 |  |
| Dementia, n (%) | 4 (1.3) | 2 (2.3) | 1 (3.2) | 0.629 |  |
| Cancer in last 3 years, n (%) | 45 (14.8) | 15 (17.4) | 5 (16.1) | 0.825 |  |
| Intracardiac thrombus/embolism, n (%) | 305 (100.0) | 86 (100.0) | 31 (100.0) | NA |  |
| Arterial embolism, n (%) | 2 (0.7) | 0 (0.0) | 0 (0.0) | 0.680 |  |
| Venous thromboembolism,  n (%) | 23 (7.5) | 9 (10.5) | 7 (22.6) | 0.020 |  |
| ACE inhibitor/ARB, n (%) | 162 (53.1) | 51 (59.3) | 14 (45.2) | 0.361 |  |
| Aspirin, n (%) | 86 (28.2) | 7 (8.1) | 6 (19.4) | <0.001 |  |
| Beta-blocker, n (%) | 106 (34.8) | 66 (76.7) | 10 (32.3) | <0.001 |  |
| Statin, n (%) | 116 (38.0) | 42 (48.8) | 11 (35.5) | 0.169 |  |
| Antiplatelet agents (excluding heparin and aspirin), n (%) | 104 (34.1) | 11 (12.8) | 6 (19.4) | <0.001 |  |
| MRA, n (%) | 3 (1.0) | 3 (3.5) | 1 (3.2) | 0.214 |  |
| Digitalis glycosides, n (%) | 1 (0.3) | 8 (9.3) | 0 (0.0) | <0.001 |  |
| Diuretics, n (%) | 61 (20.0) | 24 (27.9) | 4 (12.9) | 0.145 |  |
| Long-acting nitrates, n (%) | 18 (5.9) | 5 (5.8) | 1 (3.2) | 0.827 |  |
| Calcium-channel blocker,  n (%) | 84 (27.5) | 26 (30.2) | 5 (16.1) | 0.311 |  |
| NT-proBNP, median (IQR) | 261.00 (154.25, 478.5) | 603.50 (352.75, 783.0) | 614.50 (335.5, 1276.5) | <0.001 |  |
| BMI, mean (SD) | 26.53 (4.19) | 23.38 (7.40) | 26.07 (4.09) | 0.055 |  |

HF; Heart failure, AF; Atrial fibrillation; TIA; Transient ischemic attack, ACE inhibitor; Angiotensin-Converting enzyme inhibitor, ARB; Angiotensin II receptor blocker, MRA; Mineralocorticoid Receptor Antagonist, NT-proBNP; N-terminal pro-B-type natriuretic peptide; BMI; Body Mass Index, SD; Standard deviation, IQR; Interquartile range. Heparin and aspirin excluded in the group Antiplatelet agents. Other vascular disease: Peripheral arterial disease

| **Supplementary Table S2A. HF Hazard Ratios from Cox Models in SSII Participants, Adjusted for NT-proBNP, Weight, and Height** | | | |
| --- | --- | --- | --- |
|  |  |  |  |
| **Outcome** | **Group** | **Adjusted HR (CC on NT-proBNP/Height/Weight, n=6 144) (95% CI)** | **Adjusted HR (SSII alt, n=6 144) (95% CI)** |
| HF | No AF (ref) | 1.00 | 1.00 |
| HF | Screening-detected AF | 4.65 (3.19 - 6.79) | 2.96 (2.00 – 4.37) |
| HF | Previous AF | 1.30 (0.70 – 2.42) | 1.17 (0.63 – 2.18) |
|  |  |  |  |
| SS/SSII adjusted models include: age, female gender, coronary heart disease, hypertension, diabetes, previous stroke/TIA, chronic kidney disease, cancer, vascular disease, valvular heart disease. SS; STROKESTOP, SSII; STROKESTOP II, HF; Heart failure, AF; Atrial fibrillation, PY; Person years, CI; Confidence interval, HR; Hazard ratio, ref; reference group | | | |
| CC; Complete case, CC on NT-proBNP/Height/Weight means adjusting for all the variables above on complete cases (n= 6 144). SSII alt; Adjusted HR on all the above variables including NT-proBNP, Height, Weight in STROKESTOP II. | | | |

| **Supplementary Table S2B. HF Hazard Ratios from Fine-Gray models in SSII Participants, Adjusted for NT-proBNP, Weight, and Height** | | | |
| --- | --- | --- | --- |
|  |  |  |  |
| **Outcome** | **Group** | **Adjusted HR (CC on NT-proBNP/Height/Weight, n=6 144) (95% CI)** | **Adjusted HR (SSII alt, n=6 144) (95% CI)** |
| HF | No AF (ref) | 1.00 | 1.00 |
| HF | Screening-detected AF | 4.26 (2.82 – 6.43) | 2.79 (1.78 – 4.37) |
| HF | Previous AF | 1.34 (0.71 – 2.53) | 1.21 (0.63 – 2.30) |
|  |  |  |  |
| SS/SSII adjusted models include: age, female gender, coronary heart disease, hypertension, diabetes, previous stroke/TIA, chronic kidney disease, cancer, vascular disease, valvular heart disease. SS; STROKESTOP, SS2; STROKESTOP II, HF; Heart failure, AF; Atrial fibrillation, PY; Person years, CI; Confidence interval, HR; Hazard ratio, ref; reference group | | | |
| CC; Complete case, CC on NT-proBNP/Height/Weight means adjusting for all the variables above on complete cases (n= 6 144). SS2 alt; Adjusted HR on all the above variables including NT-proBNP, Height, Weight in STROKESTOP II. **Supplementary Table S3A. Hazard Ratios from Fine–Gray Models in Participants from SS and SSII** \| \| \|; **SS (n= 6 824)** \| \| \|; **Outcome** \| **Group** \| **HR (95% CI)** \| **Adjusted HR (95% CI)**; HF \| No AF (ref) \| 1.00 \| 1.00; HF \| Screening-detected AF \| 3.51 (2.65 – 4.64) \| 3.14 (2.35 – 4.20); HF \| Previous AF \| 4.13 (3.42 – 4.98) \| 2.82 (2.29 – 3.48); **SSII (n= 6 601)** \| \| \|; **Outcome** \| **Group** \| **HR (95% CI)** \| **Adjusted HR (95% CI)**; HF \| No AF (ref) \| 1.00 \| 1.00; HF \| Screening-detected AF \| 4.44 (3.05 – 6.46) \| 4.36 (2.92 – 6.50); HF \| Previous AF \| 3.85 (3.03 – 4.89) \| 2.62 (2.01 – 3.40); SS/SSII adjusted models include: age, female gender, coronary heart disease, hypertension, diabetes, previous stroke/TIA, chronic kidney disease, cancer, vascular disease, valvular heart disease. \| \| \|; SS; STROKESTOP, SSII; STROKESTOP II, HF; Heart failure, AF; Atrial fibrillation, PY; Person years, CI; Confidence interval, HR; Hazard ratio, ref; reference group \| \| \| **Supplementary Table S3B. Hazard Ratios from Fine–Gray Models in Controls from SS and SSII** \| \| \|; **SS (n= 12 898)** \| \| \|; **Outcome** \| **Group** \| **HR (95% CI)** \| **Adjusted HR (95% CI)**; HF \| No AF (ref) \| 1.00 \| 1.00; HF \| Previous AF \| 3.09 (2.73 – 3.51) \| 2.29 (2.00 – 2.63); **SSII (n= 12 905)** \| \| \|; **Outcome** \| **Group** \| **HR (95% CI)** \| **Adjusted HR (95% CI)**; HF \| No AF (ref) \| 1.00 \| 1.00; HF \| Previous AF \| 3.66 (3.19 – 4.20) \| 2.64 (2.27 – 3.08); SS/SSII adjusted models include: age, female gender, coronary heart disease, hypertension, diabetes, previous stroke/TIA, chronic kidney disease, cancer, vascular disease, valvular heart disease. \| \| \|; SS; STROKESTOP, SSII; STROKESTOP II, HF; Heart failure, AF; Atrial fibrillation, PY; Person years, CI; Confidence interval, HR; Hazard ratio, ref; reference group \| \| \| **Supplementary Table S4. Time-varying AF sensitivity analysis for incident HF (Cox models)** \| \| \| \| \| \| \| \|; **Population** \| **Study** \| **Model** \| **n persons** \| **n intervals** \| **HF events** \| **Screening-detected AF HR (95% CI)** \| **Previous AF HR (95% CI)** \| **Incident AF during follow-up HR (95% CI)**; **Participants** \| SS \| Unadjusted \| 6,824 \| 7,451 \| 631 \| 4.56 (3.44 – 6.04) \| 5.42 (4.45 – 6.60) \| 7.03 (5.64 – 8.78); \| \| Adjusted \| 6,824 \| 7,451 \| 631 \| 4.10 (3.08 – 5.44) \| 3.82 (3.09 – 4.71) \| 5.96 (4.76 – 7.46); \| SSII \| Unadjusted \| 6,601 \| 7,066 \| 422 \| 6.08 (4.17 – 8.85) \| 5.12 (3.99 – 6.57) \| 11.51 (8.88 – 14.92); \| \| Adjusted \| 6,601 \| 7,066 \| 422 \| 6.30 (4.32 – 9.19) \| 3.57 (2.75 – 4.64) \| 10.38 (7.98 – 13.49); **Controls** \| SS \| Unadjusted \| 12,898 \| 14,149 \| 1,429 \| \| 4.11 (3.61 – 4.67) \| 6.91 (6.02 – 7.94); \| \| Adjusted \| 12,898 \| 14,149 \| 1,429 \| \| 3.12 (2.72 – 3.57) \| 6.11 (5.31 – 7.03); \| SSII \| Unadjusted \| 12,905 \| 13,875 \| 1,050 \| \| 4.76 (4.13 – 5.48) \| 8.31 (7.03 – 9.83); \| \| Adjusted \| 12,905 \| 13,875 \| 1,050 \| \| 3.48 (2.99 – 4.05) \| 7.47 (6.31 – 8.84) Cox regression models were estimated in counting-process form, with AF handled as a time-varying exposure. Death and administrative end of follow-up were treated as censoring events. Previous AF and, among participants, screening-detected AF were defined at study index. Among individuals without AF at study index, person-time was classified as no AF until the first register-recorded AF diagnosis and as incident AF thereafter. Controls therefore contributed no-AF, previous-AF, or incident-AF person-time, but not screening-detected-AF person-time. AF recorded on the same date as the first HF event was not allowed to switch exposure status before the HF event. Adjusted models included age, sex, coronary heart disease, hypertension, diabetes, previous stroke/TIA, chronic kidney disease, cancer, vascular disease, and valvular heart disease. Among baseline no-AF individuals, the number contributing incident-AF follow-up after switching exposure status was 627 in SS participants, 465 in SSII participants, 1 253 in SS controls, and 970 in SS2 controls. AF and HF recorded on the same date, and therefore not used for exposure switching, occurred in 58, 37, 166, and 126 individuals, respectively. SS, STROKESTOP; SSII, STROKESTOP II; HF, heart failure; AF, atrial fibrillation; HR, hazard ratio; CI, confidence interval. | | | |

| **Supplementary Table S5A. Sensitivity analysis excluding incident HF within one month post-index** | | | | | | |
| --- | --- | --- | --- | --- | --- | --- |
| **Model type** | **Population** | **Study** | **Model** | **n** | **Screening-detected AF HR (95% CI)** | **Previous AF HR (95% CI)** |
| Cox | Participants | SS | Unadjusted | 6,820 | 3.60 (2.73 – 4.74) | 4.23 (3.50 – 5.12) |
|  |  |  | Adjusted | 6,820 | 3.21 (2.43 – 4.24) | 2.85 (2.33 – 3.50) |
|  |  | SSII | Unadjusted | 6,597 | 4.63 (3.20 – 6.70) | 3.85 (3.03 – 4.90) |
|  |  |  | Adjusted | 6,597 | 4.79 (3.30 – 6.94) | 2.59 (2.01 – 3.34) |
|  | Controls | SS | Unadjusted | 12,876 |  | 3.20 (2.82 – 3.63) |
|  |  |  | Adjusted | 12,876 |  | 2.33 (2.04 – 2.67) |
|  |  | SSII | Unadjusted | 12,891 |  | 3.73 (3.25 – 4.28) |
|  |  |  | Adjusted | 12,891 |  | 2.64 (2.28 – 3.05) |
| Fine-Gray | Participants | SS | Unadjusted | 6,820 | 3.53 (2.67 – 4.66) | 4.09 (3.38 – 4.95) |
|  |  |  | Adjusted | 6,820 | 3.17 (2.37 – 4.23) | 2.81 (2.28 – 3.48) |
|  |  | SSII | Unadjusted | 6,597 | 4.49 (3.08 – 6.53) | 3.85 (3.03 – 4.89) |
|  |  |  | Adjusted | 6,597 | 4.40 (2.95 – 6.58) | 2.62 (2.01 – 3.42) |
|  | Controls | SS | Unadjusted | 12,876 |  | 3.05 (2.69 – 3.46) |
|  |  |  | Adjusted | 12,876 |  | 2.27 (1.97 – 2.60) |
|  |  | SSII | Unadjusted | 12,891 |  | 3.65 (3.18 – 4.18) |
|  |  |  | Adjusted | 12,891 |  | 2.63 (2.26 – 3.07) |
| Individuals with incident HF within one month after index were excluded before model fitting. One month was operationalized as follow-up time ≤ 1/12 year among those with incident HF. | | | | | | |
| No AF is the reference group. Cox models censor deaths before HF; Fine-Gray models treat deaths before HF as competing events. | | | | | | |
| Adjusted models include age, female sex, coronary heart disease, hypertension, diabetes, previous stroke/TIA, chronic kidney disease, cancer in the last 3 years, other vascular disease, and valvular heart disease.  HF; heart failure, SS; STROKESTOP, SSII; STROKESTOP II, n; number. | | | | | | |

| **Supplementary Table S5B. Exclusions for one-month post-index HF sensitivity analysis** | | | | |
| --- | --- | --- | --- | --- |
| **Study** | **Population** | **Initial n** | **HF events within 1 month excluded** | **n after exclusion** |
| SS | Controls | 12,898 | 22 | 12,876 |
| SSII | Controls | 12,905 | 14 | 12,891 |
| SS | Participants | 6,824 | 4 | 6,820 |
| SSII | Participants | 6,601 | 4 | 6,597 |

HF; heart failure, SS; STROKESTOP, SSII; STROKESTOP II, n; number.
